# Effects of Thermal Cross-Linking on the Structure and Property of Asymmetric Membrane Prepared from the Polyacrylonitrile

**DOI:** 10.3390/polym10050539

**Published:** 2018-05-17

**Authors:** Xin Jin, Lin Li, Ruisong Xu, Qiao Liu, Linghua Ding, Yanqiu Pan, Chunlei Wang, Weisong Hung, Kueirrarn Lee, Tonghua Wang

**Affiliations:** 1 State Key Laboratory of Fine Chemicals, Carbon Research Laboratory, School of Chemical Engineering, Dalian University of Technology, 2 Linggong Road, Dalian 116024, China; jinxin925@126.com (X.J.); lilin121@dlut.edu.cn (L.L.); ruisong_xu@126.com (R.X.); peakliu0525@163.com (Q.L.); dinglh@mail.dlut.edu.cn (L.D.); clwang-chem@dlut.edu.cn (C.W.); 2 Graduate Institute of Applied Science and Technology, National Taiwan University of Science and Technology, Taipei 10607, Taiwan; wshung@mail.ntust.edu.tw; 3 R&D Center for Membrane Technology, Department of Chemical Engineering, Chung Yuan University, 200 Chung Pei Road, Taoyuan 32023, Taiwan; krlee@cycu.edu.tw

**Keywords:** polyacrylonitrile, thermal cross-linking, asymmetric membrane, property

## Abstract

Improving the thermal and chemical stabilities of classical polymer membranes will be beneficial to extend their applications in the high temperature or aggressive environment. In this work, the asymmetric ultrafiltration membranes prepared from the polyacrylonitrile (PAN) were used to fabricate the cross-linking asymmetric (CLA) PAN membranes via thermal cross-linking in air to improve their thermal and chemical stabilities. The effects of thermal cross-linking parameters such as temperature and holding time on the structure, gas separation performance, thermal and chemical stabilities of PAN membranes were investigated by Fourier transform infrared spectroscopy (FTIR), X-ray photoelectron spectroscopy (XPS), positron annihilation lifetime spectroscopy (PALS), scanning electron microscopy (SEM), thermogravimetic analysis (TGA) and gas permeation test. The thermal cross-linking significantly influences the chemical structure, microstructure and pore structure of PAN membrane. During the thermal cross-linking, the shrinkage of membrane and coalescence or collapse of pore and microstructure make large pores diminish, small pores disappear and pore volumes reduce. The gas permeances of CLA-PAN membranes increase as the increasing of cross-linking temperature and holding time due to the volatilization of small molecules. The CLA-PAN membranes demonstrate excellent thermal and chemical stabilities and present good prospects for application in ultrafiltration for water treatment and for use as a substrate for nanofiltration or gas separation with an aggressive and demanding environment.

## 1. Introduction

Membrane-based separation technology as a novel and promising separation technology has been widely used to solve the problems on resources, energy and environment due to its high efficiency, low energy consumption and eco-friendliness [1,2]. The membrane materials, which are currently used in the membrane processes such as microfiltration, ultrafiltration, reverse osmosis and gas separation for the water treatment, desalination, food and biological product purification, and air separation [3,4,5,6,7,8,9,10], are mainly the polymeric membrane materials owing to their relatively simple preparation technics and adequate perm-selectivity. However, the poor thermal and chemical stabilities of classical polymeric membranes have restricted their wide applications in the relatively high temperature or demanding and aggressive environment. Therefore, enhancing the thermal and chemical stabilities of polymeric membrane materials is very important in enlarging their applications.

Cross-linking modification, including the chemical cross-linking and thermal cross-linking, is a promising method to improve and enhance the thermal and chemical stabilities of polymeric membrane. In the cross-linking modification, many research works at present focus on the chemical cross-linking, in which the polymer reacts with the cross-linking agent in the presence of a catalyst to form a cross-linking or network structure [11,12,13,14,15,16]. For instance, Rhim [17] and Heydari [18] fabricated the cross-linked poly (vinyl alcohol) membranes by using the different cross-linking agents such as sulfosuccinic acid and fumaric acid, respectively. Beppu [19] and Shenvi [20] prepared cross-linked chitosan membranes by using glutaraldehyde and sodiumtripolyphosphate as the cross-linking agents, respectively. However, the chemical cross-linking mainly improved the properties of polymeric membranes such as the hydrophilicity and separation performance. The thermal and chemical stabilities of the membranes were not ameliorated greatly, especially for the thermal stability. Compared to the chemical cross-linking, the thermal cross-linking can obviously enhance the thermal and chemical stabilities of polymeric membrane, because the thermal rearrangement and cross-linking occur in the chain segments of polymer to form a stable three-dimensional network structure during thermal cross-linking. The oxidation cross-linking, occurring in the oxidative atmosphere at lower temperature (about 200 °C), is a promising method widely used in thermal cross-linking. Unfortunately, few research works on the thermal cross-linking membranes were reported in last decade because few polymeric materials could satisfy the structural requirement for oxidative cross-linking and the complex equipment was used for preparing the cross-linked membrane. Albo and Tsuru [21,22,23] pretreated several composite membranes derived from polyamide with different methods, and discussed temperature-induced changes in polymer structure and transport properties of composite membranes. Hou [24] and Guo [25] improved the mechanical and thermal stabilities of SPEEK membranes for fuel cells application via the thermal cross-linking. Du [26] and Song [27] fabricated the cross-linking membranes derived from polymers of intrinsic microporosity (PIMs) by oxidation cross-linking to enhance and improve the gas performance of membrane. Wind [28] and Qiu [29] prepared the thermal cross-linking membrane derived from polyimide to solve the problem of CO_2_ plasticization in natural gas separation.

Polyacrylonitrile (PAN) as a good membrane material has been used to prepare the microfiltration, ultrafiltration and gas separation membrane for the water treatment, gas separation and hemodialysis [30,31,32,33,34,35]. Besides, PAN is easy to be thermal cross-linked with oxidative medium due to the high reactivity of the cyano group [36]. Hence, PAN is a suitable material to fabricate cross-linking membrane. However, PAN is a thermoplastic polymer and the motion of macromolecular chain segment will be aggravated as the heating temperature surpasses its glass-transition temperature, in which the pore structure of membrane formed in membrane fabrication will be coalesced or collapsed during the thermal cross-linking. Therefore, maintaining the original pore structure is a big challenge during the thermal cross-linking of PAN membranes. At present, research work on the preparation of PAN cross-linking membranes [37,38] have been barely reported, especially those that investigated and discussed the effects of cross-linking parameters on the chemical, pore and microstructure, transport properties of PAN membranes.

In this research work, thermal cross-linking was used to improve the thermal and chemical stabilities of PAN asymmetric membrane, which was prepared by dry-wet phase inversion technique. The motivation is to systematically investigate and discuss the effects of thermal cross-linking parameters such as temperature and holding time on the properties of PAN membranes, including the chemical, pore and microstructure, gas separation performance, thermal and chemical stabilities. The results derived here are expected to help us gain an insight into the structural changes of asymmetric PAN polymeric membranes during thermal cross-linking and find the way to keep well the asymmetric pore structure of PAN membranes in thermal cross-linking with enhancing the chemical and thermal stabilities of PAN membranes. These works will be beneficial to prepare the cross-linking PAN membrane with a well-kept pore structure and good chemical and thermal stabilities, which present a good prospect for application in ultrafiltration for water treatment and for use as a substrate for nanofiltration or gas separation with an aggressive and demanding environment.

## 2. Materials and Methods

### 2.1. Materials

Polyacrylonitrile (PAN) (181315, *M_n_* = 170,000), *N*-Dimethylacetamide (DMAc), *N*-methyl-2-pyrrolidone (NMP) and dimethylsulfoxide (DMSO) solvents were purchased from Sigma-Aldrich (Hamburg, Germany), Tianjin Fuyu Fine Chemical Co., Ltd. (Tianjin, China) and Damao Chemical Reagent Factory (Tianjin, China), respectively. Bovine serum albumin (BSA, *M_n_* = 67,000), polyethylene glycols (PEG, *M_n_* = 10,000, 20,000), ethanol and tetrahydrofuran (THF) were obtained from Newprobo Bio-Tech Co., Ltd. (Beijing, China) and Tianjing Guangfu Fine Chemical Research Institute (Tianjin, China), respectively. Chloroform (CHCl_3_) and sulfuric acid (H_2_SO_4_), Sodium hydroxide (NaOH) were purchased from Tianjin third Chemical Reagent Factory (Tianjin, China) and Xilong Chemical Co., Ltd. (Shantou, China), respectively. The deionized water was manufactured in house by a water purification device.

### 2.2. Preparation of Cross-Linked PAN Asymmetric Membranes

Dry-wet phase inversion technique was used to prepare the PAN asymmetric membranes. The homogeneous casting solution prepared by dissolving PAN powder into DMAc with continuous stirring was cast onto a glass plate. Then the cast membrane was evaporated in air for 30 s before been immersed in water to exchange the solvent for 1 day, prior to drying in air for 2 days.

**Figure 1:**
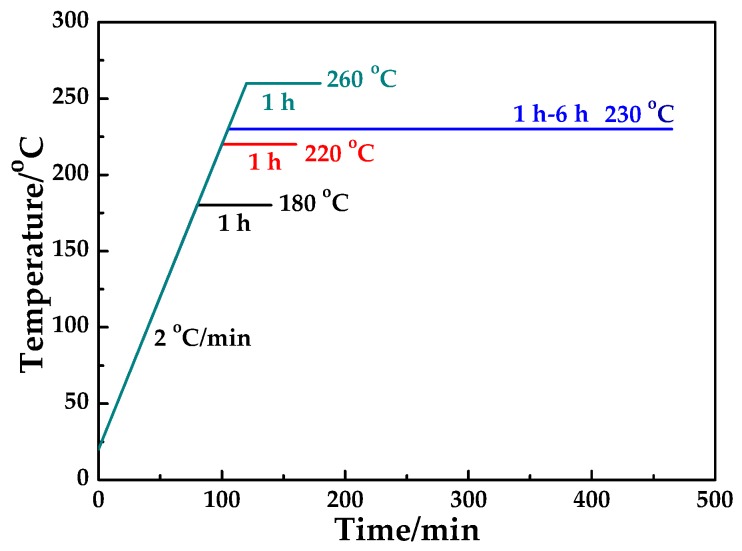
The cross-linking program of polyacrylonitrile (PAN) membrane.

The thermal cross-linking of PAN membranes was carried out in a tubular furnace. Figure 1 showed the cross-linking program. In order to investigate the effect of cross-linking temperature on the properties of PAN membranes, the different cross-linking temperatures of 180 °C, 220 °C, 230 °C and 260 °C were selected at heating rate of 2 °C/min with the holding time of 1 h and airflow rate of 500 mL/min. Holding time was kept for 1 h–6 h at 230 °C to investigate the effect of cross-linking holding time on the properties of PAN membranes. The furnace was cooled to room temperature naturally after cross-linking.

### 2.3. Characterization of Membranes

The chemical structure of PAN and CLA-PAN membranes was detected by Attenuated total reflection-Fourier transform infrared spectra (ATR-FTIR) (NICOLET 6700, Thermo Fisher Scientific, Waltham, MA, USA) and the ESCALab220i-XL X-ray photoelectron spectroscopy (XPS, Thermo Fisher Scientific, Basingstoke, UK). In order to better understand the kinetics of cross-linking, the extents of reaction (EOR) following the cross-linking temperature and holding time was introduced to quantitatively show the degree of cross-linking, which was derived from the intensity of the nitrile absorption and the absorption at 1600 cm^−1^ in the spectra of ATR-FTIR, and the EOR was calculated according to the following equation [39]:

(1) $$EOR=\frac{I_{1600}}{I_{CN}+I_{1600}}$$

where *I_CN_* and *I*_1600_ were the intensity of the nitrile absorption and the absorption at 1600 cm^−1^, respectively.

The microstructure of the as-made CLA-PAN membranes was detected and measured by TF30 transmission electron microscope (TEM, FEI, Hillsboro, OR, USA) and D/Max–2400 X-ray diffraction (XRD, Rigaku Industrial Corporation, Osaka, Japan). The cross-section morphologies of the PAN asymmetric membranes were observed by the NOVA Nano SEM 450 scanning electron microscope (SEM, FEI, Hillsboro, OR, USA).

Positron annihilation lifetime spectroscopic (PALS) experiments were conducted to determine the free-volume in PAN. A conventional fast-fast coincidence spectrometer with a time resolution of 250 ps was used. A radioactive source of ^22^Na (0.74 MBq), which was sealed in between 7.5-μm thick Kapton^®^ Type HN sheets from DuPont Inc. (Calgary, AB, Canada), was sandwiched in two stacks of membrane samples that consisted of several layers of free-standing PAN. Each stack of sample had a total thickness of 1 mm. Positron annihilation lifetimes were recorded using a fast-fast coincidence timing system. A time-to-amplitude converter was used to convert lifetimes and to store timing signals in a multi-channel analyzer (Ortec System, AMETEK ORTEC Inc., Oak Ridge, TN, USA). Two million counts were collected, and all positron annihilation lifetime spectra were analyzed by a finite-term lifetime analysis method using PATFIT and MELT programs [40,41,42].

The membrane pore sizes were also characterized using the method of the molecule weight cut-off (MWCO) by testing the flux and rejection rate of PEG 10000, PEG 20000 and BSA on a dead-end membrane module for PAN membranes and CLA-PAN membranes. During the measurement, membranes were pretreated under 0.9 MPa for 30 min, and permeated the solution of PEG 10000 and PEG 20000 (100 mg/L) and bovine serum albumin (BSA, 1 g/L) under 0.8 MPa at ambient temperature. The rejection R was calculated using the following equation:

(2) $$R=\left(1-\frac{C_{p}}{C_{f}}\right)\times 100\%$$

where *C_p_* and *C_f_* were the concentration of the permeate solution and feed solution, respectively. All the experiments on rejection were repeated for three times [43].

Permeances of O_2_/N_2_ mixtures (21/79 mol. %) for the PAN membranes and CLA-PAN membranes were measured by the variable volume-constant pressure method [44] and represented in GPU (1 GPU = 10^−6^ cm^3^ (STP)/cm^2^ s cmHg), in which the permeated gas at a feed pressure of 5 kPa at 298 K was analyzed by gas chromatography (GC, Zhejiang Fuli analytical instruments Co., Ltd., Wenling, China, GC 7890). The detailed information about the gas permeating test process was in the reference [45].

The thermal stabilities of the PAN membrane and CLA-PAN membranes were detected by the NETZSCH TG 209C thermogravimetic analyzer (TGA, Netzsch, Bayern, Germany) in nitrogen. The chemical stabilities of CLA-PAN membranes were tested by the solvent dissolution, in which the membranes were first soaked in different solvents for 4 days and then washed by water. The residual mass fraction *W* was calculated from the following equation after dried in vacuum,

(3) $$W=\left(\frac{w_{t}}{w_{0}}\right)\times 100\%$$

where *w*_0_ was the membrane mass before soaked in solvent; *w_t_* was the membrane mass after soaked in solvent, washed by water and dried in vacuum.

## 3. Results and Discussions

### 3.1. Chemical Structure Variations of PAN Asymmetric Membrane during Cross-Linking

**Figure 2:**
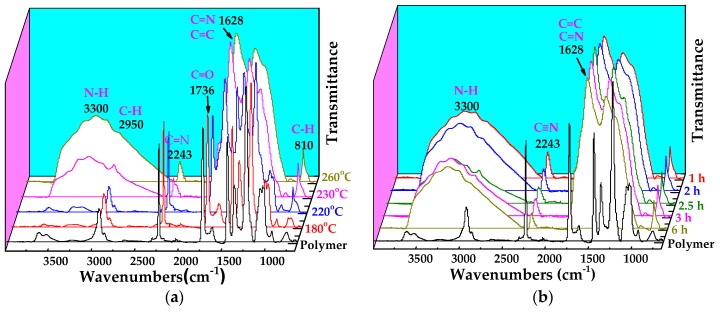

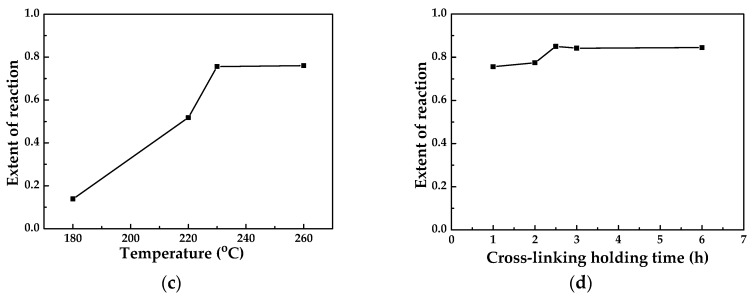
ATR-Fourier transform infrared spectroscopy (FTIR) spectra of the PAN and cross-linking asymmetric (CLA)-PAN membranes obtained at different cross-linking temperatures with the holding time of 1 h (**a**) and the extents of reaction (EOR) data of PAN membranes (**c**). ATR-FTIR spectra of the PAN and CLA-PAN membranes obtained at different cross-linking holding time with the temperature of 230 °C (**b**) and the EOR data of PAN membranes (**d**).

**Figure 3:**
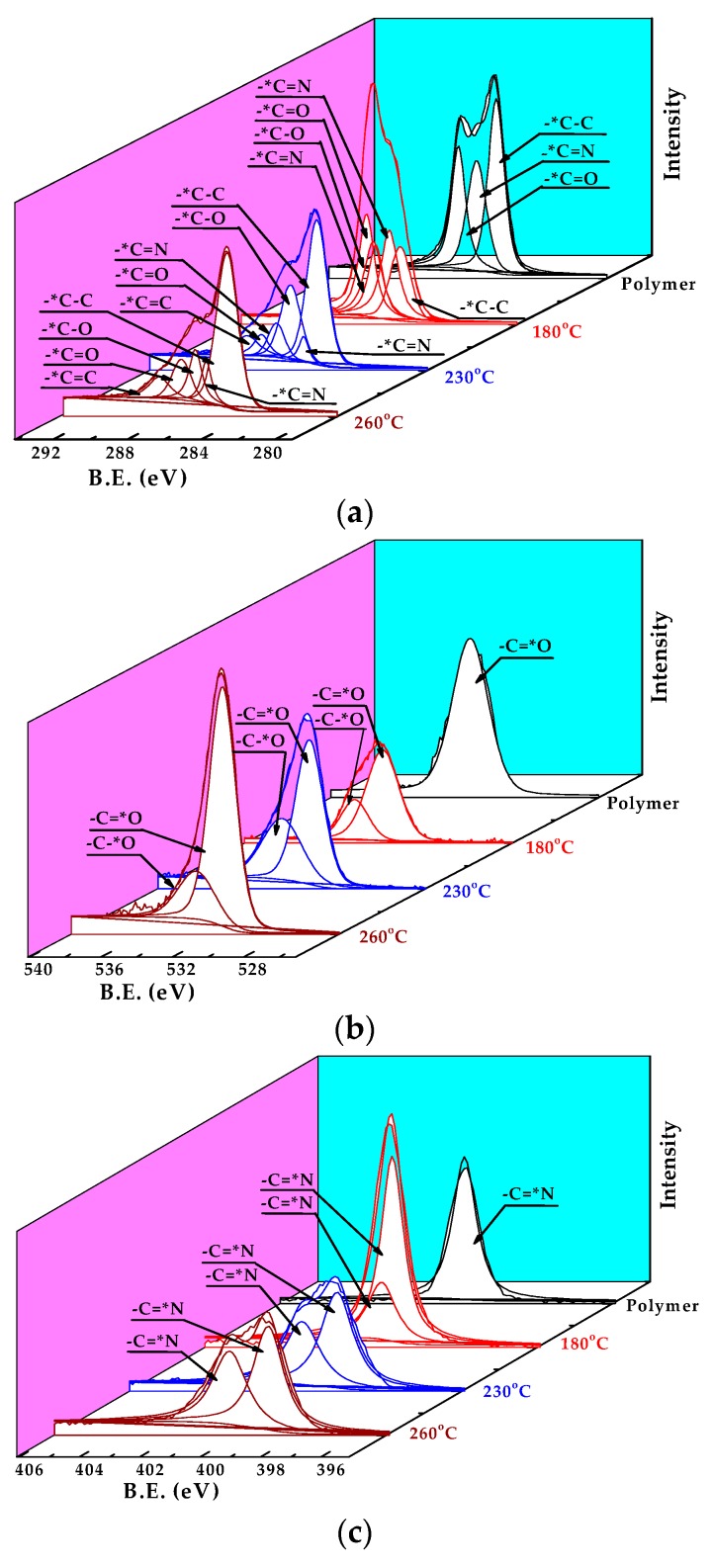
The C1s (**a**), O1s (**b**) and N1s (**c**) core levels of polymer membrane and CLA-PAN membranes prepared at different cross-linking temperatures.

**Figure 4:**
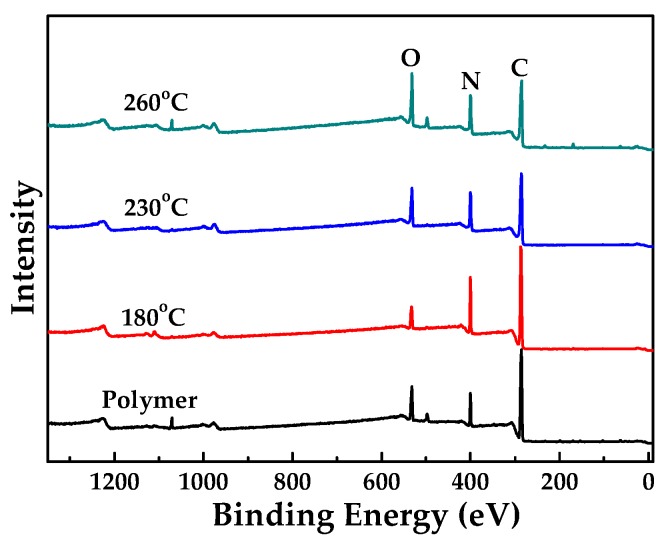
XPS spectra of the PAN and CLA-PAN membranes obtained at different cross-linking temperatures.

Figure 2 shows the ATR-FTIR spectra of PAN membrane and CLA-PAN membranes cross-linked at different temperatures and holding time. Compared to the spectra of PAN membrane, in which the typical characteristic peaks of PAN molecular are identified as –C≡N stretching vibration peak (2243 cm^−1^), –C–H stretching vibration peak (2950 cm^−1^) and C–H deformation vibration peak (810 cm^−1^), the intensity of –C≡N and –C–H stretching vibration peaks in the CLA-PAN membranes are reduced gradually, and the intensity of the –C–H deformation vibration peak is enhanced with the increase of thermal cross-linking temperature and holding time. Additionally, the –C=C and –C=N stretching vibration peak (1628 cm^−1^), –N–H stretching vibration peak (3300 cm^−1^) and C–H deformation vibration peak of C=C–H (810 cm^−1^) are observed. These indicate that –C≡N of the PAN molecular structure is ruptured and the –C=N and –N–H are formed during the thermal cross-linking. EOR data that can quantitatively show the degree of cross-linking were calculated from the intensity of the peak at 1628 cm^−1^, *I*_1600_, and that of the nitrile peak at 2243 cm^−1^, *I_CN_*, are shown in Figure 2c,d. With the increase of cross-linking temperature and holding time, the EOR data increases from 0.14 at 180 °C (1 h) to 0.85 at 230 °C (2.5 h), indicating that the cross-linking degree of PAN membranes gradually increases during the thermal cross-linking. It can also be proved from the XPS spectra of PAN membrane and CLA-PAN membranes shown in Figure 3. In the CLA-PAN membranes, new peaks of –C=N, –C=C and –C–O appear and the intensity of –C≡N peak gradually decrease in the C1s spectrum, and the intensity of –C=O and –C=N peak in O1s and N1s spectra gradually increase as the cross-linking temperature rises, suggesting that –C≡N in PAN decompose and react with oxidative medium to form the –C=N, –C=C, –C–O and –C=O. In addition, the XPS spectra of the PAN and CLA-PAN membranes (Figure 4) show that the O atomic percentage in the membrane increases with the enhancement of cross-linking temperature, indicating that O_2_ has participated in the oxidative cross-linking reaction.

**Figure 5:**
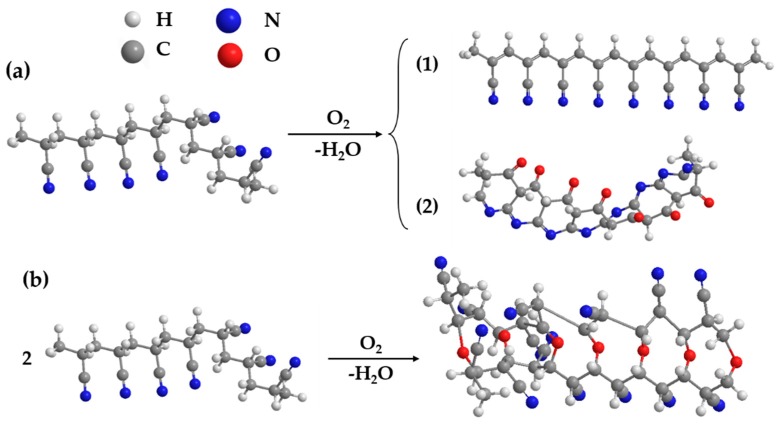
Cross-linking mechanism of PAN membrane (**a**) Intramolecular cyclization; (**b**) Intermolecular cross-linking.

According to above evidence, the oxidative cross-linking reaction of PAN in air can be speculated as following: the strong polarity of cyano groups in PAN molecule chains makes its α-H more active, and results in that the dehydrogenation more easily reacts to form the C=C among the cross-linking reaction [46]. Figure 5 illustrates the thermal cross-linking mechanism of PAN membrane. The β-C in PAN chains is oxidized to carbonyl, and –C≡N is cyclized to form –C=N [47]. Besides the intramolecular cross-linking reaction, the intermolecular cross-linking reaction might occur between the β-C of the two PAN chains that combine with O to form C–O–C structure, and finally forms a three-dimensional cross-linked network structure with thermal resistance.

### 3.2. Microstructure Variations of PAN Asymmetric membrane during Cross-Linking

**Figure 6:**
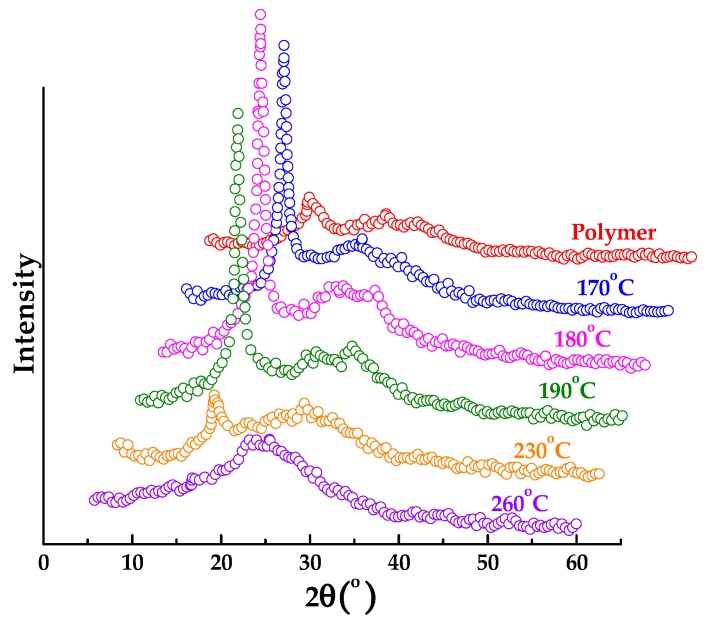
The XRD patterns of PAN membranes prepared at different cross-linking temperatures with the holding time of 1 h.

**Figure 7:**
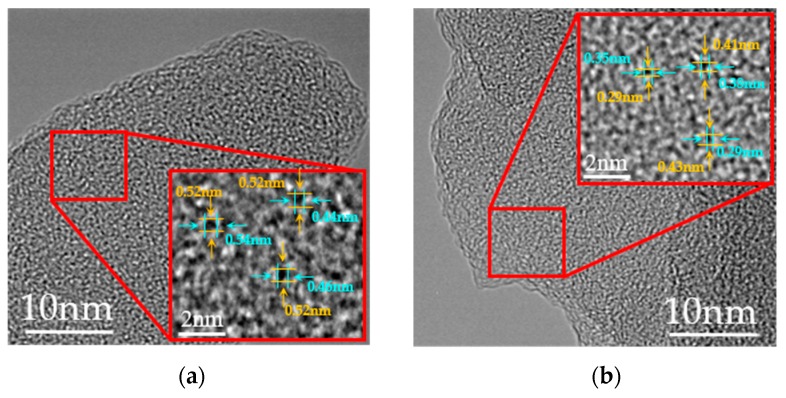
TEM images of CLA-PAN-230 (**a**) and CLA-PAN-260 (**b**) membranes.

Figure 6 presents the XRD patterns of PAN and CLA-PAN membranes prepared at different cross-linking temperatures. The obvious diffraction peak corresponding to the (100) plane of the quasi-hexagonal lattice and another weak diffraction peak corresponding to the (110) crystallographic plane appear at the 2θ value of 17.7° and 25.2°, respectively. As the cross-linking temperature increases, the intensity of (100) diffraction peak firstly increases, and then decreases. It reaches the maximum at 180 °C and disappears at 260 °C. Additionally, the intensity of (110) diffraction peak enhances with the increase of cross-linking temperature. This implies that the crystal structure of PAN membrane become ordered at the initial stage of cross-linking process, and then gradually transforms into a disordered structure as the heating temperature rises. It can be further confirmed by TEM image shown in Figure 7. The microstructure of CLA-PAN membranes prepared at 260 °C (CLA-PAN-260) looks more disordered and denser than that of membranes prepared at 230 °C (CLA-PAN-230). The measured lattice distance of the microstructure reduces from 0.46–0.5 nm to 0.37–0.38 nm as the cross-linking temperature increases from 230 °C to 260 °C, which indicated that the thermal cross-linking would make microstructure of membranes more disordered and compact at a high temperature due to the structure shrinkage of membranes.

### 3.3. Pore Structure Variations of PAN Asymmetric Membrane during Cross-Linking

**Figure 8:**
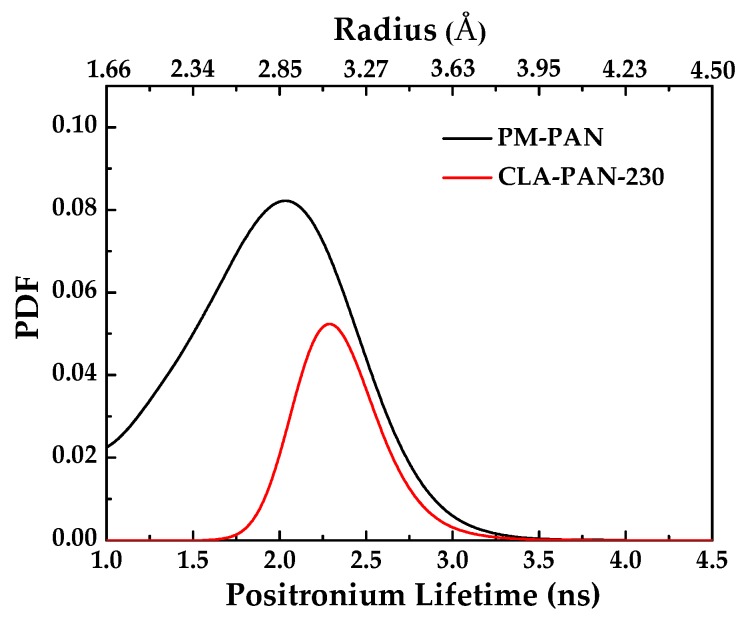
o-Ps lifetime distribution data for PAN and CLA-PAN-230 membranes.

The Table 1 and Table 2 and Figure 8 present the pore structure variations of the PAN asymmetric ultrafiltration membrane before and after thermal cross-linking, which indicate that the thermal cross-linking greatly affects the pore structure of the PAN ultrafiltration membrane.

Table 1 shows the fluxes and rejection rates of PEG 10000, PEG 20000 and BSA for PAN membrane and CLA-PAN membranes, which reflect the changes of pore structure larger than 1 nm. Compared to the PAN membrane, the fluxes of CLA-PAN membranes obviously decrease and the rejection rates increase, which suggest that the pore volumes of PAN membrane reduce and pore sizes (>1 nm) become smaller during thermal cross-linking.

PALS experiments are normally used to determine the free-volume that size is less than 1 nm in polymeric membranes. Table 2 presents the free-volume of the PAN membrane and CLA-PAN membranes measured by PALS, in which the o-Ps lifetime (τ_3_) and the relative intensity (I_3_) correspond to the size and the concentration of the free volume, respectively. Compared to the PAN membrane, CLA-PAN membrane has a lower I_3_, bigger τ_3_ and R_3_, which indicates that the concentration of free volume is reduced and the average free volume radius of PAN membrane is largened after thermal cross-linking. Figure 8 shows the o-Ps lifetime distribution of PAN membrane and CLA-PAN membrane. After thermal cross-linking, the free volume concentration of PAN membrane diminishes and free volume distribution of PAN membrane becomes narrow. The free volumes which sizes are below 0.25 nm are vanished. That all suggests that the pore structure of PAN ultrafiltration membrane is obviously changed during thermal cross-linking. The large pores diminish, small pores disappear and pore volumes reduce.

**Figure 9:**
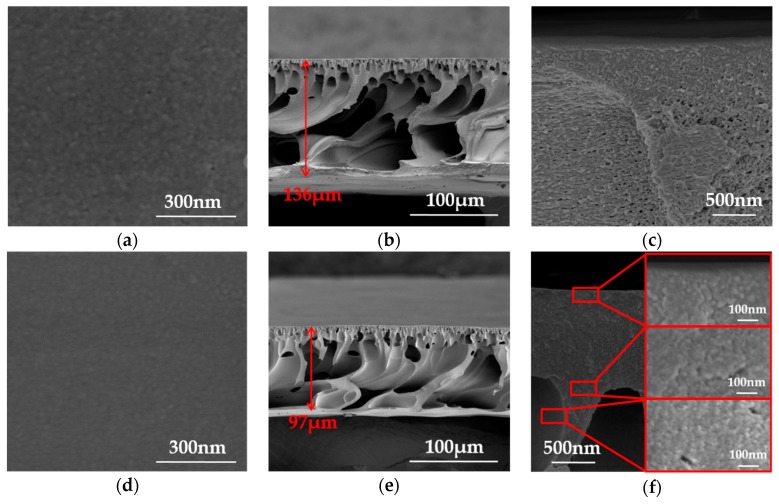
The SEM images of PAN membrane (**a**–**c**) and CLA-PAN-230 membrane (**d**–**f**) (**a**,**d**: surface; **b**,**e**: cross-sectional; **c**,**f**: enlargement of the dense layer).

Figure 9 shows the SEM images of the PAN and CLA-PAN membrane. PAN membrane possesses a typical asymmetric structure with a dense top skin layer followed by the sponge-like and finger-like porous sub-layers. After thermal cross-linking, the thickness of the membrane reduces from 136 μm to 97 μm. The large pores of membrane such as the finger-like holes and some large sponge-like pores are well maintained, and some smaller sponge-like pores disappear. On the top surface of dense skin layer, some pores diminish and some pores seem to vanish after thermal cross-linking, which hints that the coalescence or collapse of pore and microstructure occurs in the CLA-PAN-230 membrane besides the shrinkage of membrane during thermal cross-linking at the temperature of 230 °C. According to SEM images and results obtained from Table 1 and Table 2 and Figure 8, it can be concluded that the changes of pore structure of PAN ultrafiltration membrane during thermal cross-linking are attributed to the shrinkage of membrane and the coalescence or collapse of pore and microstructure. The shrinkages of membranes make the large pores smaller and the coalescence or collapse of pore and microstructure might form the dead-end pores or make the small pores disappear which will significantly affect the pore structure of derived CLA-PAN membranes, and even destroy the pore structure. Hence, controlling or reducing the coalescence or collapse of pore and microstructure during thermal cross-linking will be very important in the preparation of thermal cross-linking membrane with well-kept pore structure.

### 3.4. Gas Separation Performance of PAN and CLA-PAN Membranes

**Figure 10:**
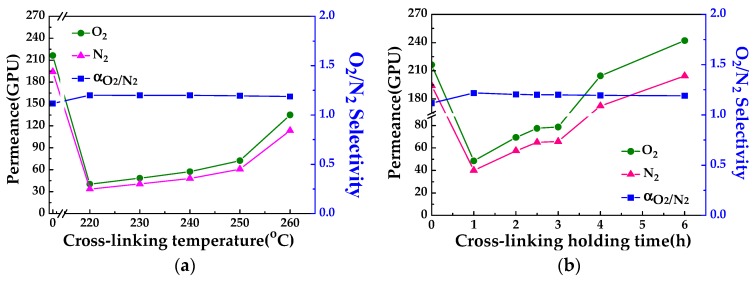
O_2_ and N_2_ mixed gas permeances of PAN and CLA-PAN membranes prepared at different cross-linking temperatures (**a**) and in different cross-linking holding time (**b**).

The mixed gas performances of PAN and CLA-PAN membranes are investigated (shown in Figure 10 and Table 3) to further understand the changes of pore size and structure in the PAN membrane during the cross-linking. Compared to the PAN membrane, the gas permeance of CLA-PAN membrane reduces obviously, and the O_2_/N_2_ selectivity slightly increases due to the shrinkage of membrane and the coalescence or collapse of pore and microstructure during cross-linking. As the cross-linking temperature increases (Figure 10a), gas permeance of CLA-PAN membrane enhances and O_2_/N_2_ selectivity keeps constant (about 1.2), which is slightly higher than the value of Knudsen diffusion (0.935). And as the cross-linking holding time prolongs (Figure 10b), gas permeance enhances more obviously and the O_2_/N_2_ selectivity slightly decreases. The O_2_ permeance of the asymmetric membrane is 242 GPU at the cross-linking holding time of 6 h at 230 °C, which is higher than the PAN membrane (shown in Table 3). It might be attributed to formation of new gas penetrating channels created by the escape of small molecules existing in the membrane matrix such as solvent molecules and unstable functional groups during cross-linking at higher temperature and longer holding time. However, the channels formed in membrane matrix can only act as the gas molecular penetrating routes and cannot contribute to improving the filtration of PEG molecule larger than 10,000 Da (Table 1). That suggests that the change of pore structure of PAN ultrafiltration membrane during thermal cross-linking are attributed not only to the shrinkage of membrane and the coalescence or collapse of pore and microstructure but also to the new gas penetrating channels formed in membrane matrix during the cross-linking. In addition, it must be noted that the poor gas selectivity of the derived CLA-PAN membranes suggests that they are not suitable for gas separation but can be used as a substrate for preparing a composite gas separation membrane.

### 3.5. Thermal Stability of PAN and CLA-PAN-230 Membranes

**Figure 11:**
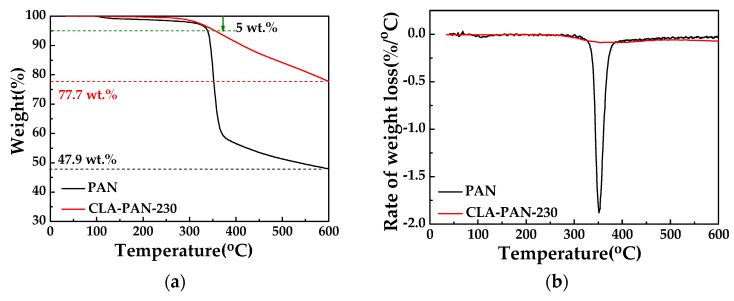
The TGA (**a**) and DTG (**b**) curves of PAN membrane and CLA-PAN-230 membrane.

The thermal stabilities of the PAN and CLA-PAN-230 membranes were investigated via TGA (Figure 11). As the temperature rises at higher than 300°C, the PAN membrane shows a sharp weight loss, and the CLA-PAN-230 membrane is gradually declining (in Figure 11a). The PAN membrane reveals a maximum weight loss rate at the temperature between the 320 °C–390 °C, and the CLA-PAN-230 membrane cannot observed it clearly (in Figure 11b). Suggesting that strong thermal decomposition reaction occurs in the PAN membrane during pyrolysis, but in the CLA-PAN membrane, it seems not to happen during pyrolysis due to its cross-linking structure, which indicates that the thermal cross-linking greatly improves the thermal stability of PAN membrane [50]. Commonly, the temperature at 5% weight loss and weight remaining (char yield) at 600 °C are used to evaluate the thermal stability of membranes at the early stage and the final stage of pyrolysis, respectively [34]. For the CLA-PAN-230 membrane, the temperature at 5% weight loss and the char yield are 354.5 °C and 77.7%, respectively. They are higher than those of PAN membrane (338.5 °C and 47.9%), indicating that the thermal stability of CLA-PAN membranes is improved. It means that the thermal cross-linking greatly enhances the thermal stability of PAN membrane.

### 3.6. Chemical Stability of PAN and CLA-PAN Membranes

**Figure 12:**
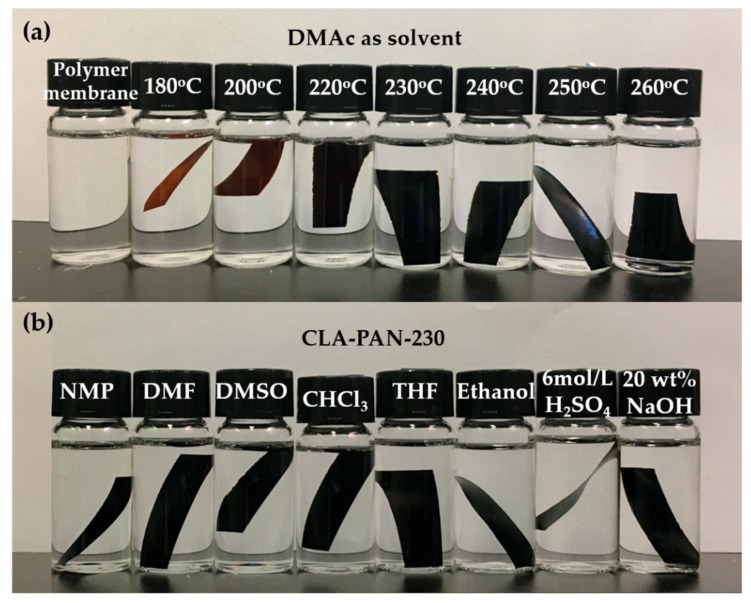
Solubility of PAN and CLA-PAN membranes prepared at different cross-linking temperatures in N-Dimethylacetamide (DMAc) (**a**); solubility of CLA-PAN-230 membrane in common solvents (**b**).

The solubility of membranes in DMAc was commonly adopted to characterize the solvent resistance or chemical stability of the membrane materials, because DMAc is a strong polar solvent. Figure 12 and Table 4 exhibit the solubility of CLA-PAN membranes in DMAc. The solubilities of CLA-PAN membranes in other solvents are also shown in Table 5. Compared to PAN membrane, which is completely dissolved in DMAc (Figure 12), the solubilities of CLA-PAN membranes decline rapidly as the increase of cross-linking temperature. The mass residual rates of CLA-PAN membranes cross-linked above 230 °C are 100%, which means that the CLA-PAN membranes are insoluble in DMAc (Table 4). Furthermore, results shown in Table 5 demonstrate that CLA-PAN-230 membrane is also insoluble in NMP, DMF, DMSO, CHCl_3_, THF, alcohol, H_2_SO_4_ (6 mol/L) and NaOH (20%). These indicate that the PAN membrane exhibits a strong chemical resistant stability after cross-linking.

## 4. Conclusions

The CLA-PAN membranes with favorable thermal and chemical stabilities were fabricated via thermal cross-linking in air. The thermal cross-linking parameters such as temperature and holding time obviously affect the structure and the properties of CLA-PAN membranes, including the chemical and microstructure, pore structure and gas separation performance of membrane. During the cross-linking, the –C≡N in the structure of PAN molecular ruptures to form the –C=N and –N–H, and O_2_ participates the reaction and forms the cross-linking structure. The crystal structure of the PAN membrane firstly becomes ordered; and then transforms into a disordered structure with the increase of the cross-linking temperature. The pore structure of PAN membrane obviously changed during thermal cross-linking. The large pores diminish, small pores disappear and pore volumes reduce, which attributed to the shrinkage of membrane and the coalescence or collapse of pore and microstructure. Controlling or reducing the coalescence or collapse of pore and microstructure during thermal cross-linking will be very important in the preparation of thermal cross-linking membrane with well-kept pore structure. The gas permeance of CLA-PAN membranes increases gradually with a steady selectivity as the increase of the cross-linking temperature and holding time. In addition, the CLA-PAN membranes exhibit good thermal and chemical stabilities. They are insoluble in various solvents, even though in the strong solvents such as DMAc and NMP. Results derived in this work demonstrate that CLA-PAN membranes have a good prospect for application in ultrafiltration for water treatment and for use as a substrate for nanofiltration or gas separation with an aggressive and demanding environment.

## Figures and Tables

**Table 1:** Fluxes and rejection rates of PAN membrane, CLA-PAN-230 and CLA-PAN-260 membranes.

Membrane	PEG 10000		PEG 20000		BSA	
	Flux (L·m^−3^ h^−1^)	Rejection Rate (%)	Flux (L·m^−3^ h^−1^)	Rejection Rate (%)	Flux (L m^−3^ h^−1^)	Rejection Rate (%)
PAN	13.76	10.35	11.08	44.16	8.23	99.26
CLA-PAN-230	0.42	14.47	0.33	52.32	0.17	99.9
CLA-PAN-260	0.31	18.71	0.24	59.93	0.10	100
Pore size (nm)	~1.8 [48]		~2.4 [48]		~6.8 [49]	

**Table 2:** o-Ps lifetime, relative intensity, and average free-volume radius data for PAN and CLA-PAN-230 membrane.

Membrane	τ_3_^1^ (ns)	I_3_^2^ (%)	R_3_^3^ (Å)
PAN	2.1138	9.0862	2.9576
CLA-PAN-230	2.3773	3.2015	3.1790

^1^ τ_3_: o-Ps lifetime corresponding to free-volume size of membrane; ^2^ I_3_: o-Ps intensity corresponding to free-volume concentration of membrane; ^3^ R_3_: average free-volume size of membrane.

**Table 3:** Mixed gas separation performances of PAN membrane and CLA-PAN membranes.

Membrane	Mixed Gas Permeance		Selectivity
	O_2_ (GPU)	N_2_ (GPU)	O_2_/N_2_
PAN	216	194	1.11
CLA-PAN-230-1 h	49	40	1.22
CLA-PAN-230-6 h	242	205	1.18
Knudsen diffusion			0.935

**Table 4:** The mass residual of PAN and CLA-PAN membranes prepared at different cross-linking temperatures dissolved in DMAc.

Cross-Linking Temperature (°C)	Polymer Membrane	180	200	220	230	240	250	260
Mass residual (wt %)	0	95.34	97.47	99.18	100	100	100	100

**Table 5:** The mass residual of CLA-PAN-230 membrane dissolved in common solvents.

Solvent	NMP	DMF	DMSO	CHCl_3_	THF	Ethanol	6 mol/L H_2_SO_4_	20 wt % NaOH
Mass residual (wt %)	100	100	100	100	100	100	99.99	100
